# Supplementary material for: Factors associated with modern contraceptives uptake during the first year after birth in Ethiopia: A systematic review and meta-analysis
Source: PLoS One. 2023 Feb 7;18(2):e0270055. doi: 10.1371/journal.pone.0270055 (PMC9904466; doi:10.1371/journal.pone.0270055)
Supplement: S1 File — (PDF) [file pone.0270055.s001.pdf]

To enable PROSPERO to focus on COVID-19 submissions, this registration record has undergone basic automated checks for eligibility and is published exactly as submitted. It has since been amended by the author and the PROSPERO team have checked the record for eligibility. PROSPERO has never provided peer review, and usual checking by the PROSPERO team does not endorse content. Therefore, automatically published records should be treated as any other PROSPERO registration. Further detail is provided here.

Citation

Gebi Husein Jima. What factors are associated to postpartum family planning utilization in Ethiopia?: a systematic review and meta-analysis. PROSPERO 2020 CRD42020159470 Available from:
https://www.crd.york.ac.uk/prospero/display_record.php?ID=CRD42020159470

Review question

What factors are affecting the uptake of family planning method during postpartum period in Ethiopia?

What is the level of postpartum family planning in Ethiopia?

Searches

In our search we will include all published and unpublished studies conducted before November 30, 2019. Studies written in English language will only be included. We will use both Electronic sources and Non-electronic/other applicable
sources. We will be using common database searches such as PubMed (MEDLINE), CNIHAL (EBSCO), Embase, google and Google Scholar. During searching foe relevant studies we will be using MeSH terms, key terms.
For non-electronic sources, we will use unpublished papers, grey literature sources and unpublished studies.

Types of study to be included

Observational studies: cross-sections, comparative cross-sections, matched and unmatched case control, prospective and retrospective cohort studies conducted on factors associated to postpartum family planning uptake in Ethiopia will be included. Both published and unpublished studies will be used. This is because observational studies are the most common types of studies available in Ethiopia. Intervention studies like randomized control trial are less likely available in Ethiopia.

Condition or domain being studied

Factors affecting uptake of postpartum family planning method among women of reproductive age group in Ethiopia

Participants/population

Women in reproductive age in Ethiopia

Intervention(s), exposure(s)

Family planning methods uptake during postpartum period versus educational level of women, knowledge/awareness level of women about postpartum family planning, Residence of women(Rural/Urban), Counseling on Postpartum family planning method at antenatal and postnatal service delivery sites, use of antenatal and postnatal service during the previous pregnancy and partner discussion on postpartum family planning method. We will also include other relevant variables based on the review result

Comparator(s)/control

Women not using family planning methods during postpartum period

Context

Studies conducted in any part of Ethiopia will be included

Main outcome(s)

Family planning methods use during postpartum period

Measures of effect

Effect measures are pooled Odds Ratios

Additional outcome(s)

no additional outcome(s)

Measures of effect

no additional outcome(s)

Data extraction (selection and coding) [1 change]

All searches of the electronic databases will be conducted using MeSH terms and relevant key terms. We will first thoroughly read the title of each considered articles/research reports, then the abstract. Then research questions and then full body of each article/research reports. Two independent reviewers (GHJ and MKA) will do the whole process. Each and every considered article/research reports will be evaluated as per the PRISMA-P (2013) guidelines.
The titles of the article/paper clearly indicating predictors/factors associated/factors affecting/factors influencing/factors related/determinants/determinant factors/risk factors/ barriers/ of postpartum family planning methods adoption/uptake/utilization in Ethiopia will be identified for strict evaluation as per the checklist.
All identified articles based on their titles will be carefully evaluated by reading the full body of the article/research reports: sections like background, objectives/research questions/hypothesis, methods and materials sections. In addition, results/findings of the studies related to factors associated to postpartum family planning utilization. Multivariate results related tables reporting adjusted odds ratio with 95% confidence interval and p-value will be given attention in the process of articles/research report evaluation. Studies not eligible based on fully paper evaluation will be excluded (reasons will be documented). If there is disagreement between the two reviewers on the decision about the inclusion/exclusion of papers evaluated, a third experienced experts (will be using the same checklist for evaluation) will be involved to decide and that will be taken as a final decision on that specific article/research report.
Important settings and findings from the finally included articles/research reports will be extracted by two independent reviewers (GHJ and MKA) using a data extraction templates. From each included studies, all required data will be extracted and summarized using temples dedicated for this purpose. These templates will be first prepared and used on Microsoft Excel, then copied to Microsoft word (2016) for presentation as part of systematic review report.

Risk of bias (quality) assessment

We will carefully evaluate quality of the included articles using the criteria/checklist which will be prepared for this purpose. All published and not published articles/research reports will be used. All recommended data sources will be searched.

Methodological related quality of each included article will be evaluated using the AMSTAR methodology assessment tool.

Joint working schedule will be prepared and strictly used. The whole review process will be as per this schedule.

Strategy for data synthesis

Data synthesis and statistical analysis will be conducted using Review Manager (RevMan) version 5.3.All the selected studies will carefully added to the RevMan.Outcomes and important comparisons will be carefully added to the software. All extracted quantitative date (Sample size(n) , number of women using and not using postpartum family planning for each considered predictors/factors associated/factors affecting/factors influencing/factors related/determinants/determinant factors/risk factors/ barriers/ of postpartum family planning methods uptake/adoption/utilization will be will be carefully and strictly added to the RevMan.

Heterogeneity between the included studies will be evaluated using the I^2 statistic(the interpretation of the heterogeneity level will be seen per the recommended values of I^2).
Symmetry of generated funnel plot and Egger's regression we will be visualized for potential publication bias(publication bias will be assumed for P-values of less than 0.10.). To compute a pooled estimate, a meta analysis will be carried out: if the variability among the studies is low.But if Heterogeneity is beyond the recommended value of I^2 , then we will descriptively report the results .

Analysis of subgroups or subsets

We will carryout subgroup analysis specially when heterogeneity between reviewed papers is high with regards to factors affecting postpartum family planning uptake

Contact details for further information

Gebi Husein Jima
gebihussein@gmail.com

Organisational affiliation of the review

Arsi University
www.arsiun.edu.et

Review team members and their organisational affiliations [1 change]

Mr Gebi Husein Jima. Arsi University

Collaborators

Mr Hailu Fekadu Demissie. Arsi University

Type and method of review

Systematic review

Anticipated or actual start date [1 change]

16 April 2021

Anticipated completion date [1 change]

16 October 2021

Funding sources/sponsors

Mr. Hailu Fekadu, Arsi university college of health science, research and community service vice dean.
Email: hailufekadu18@yahoo.com

Conflicts of interest

Language
English

Country
Ethiopia

Stage of review
Review Ongoing

Subject index terms status
Subject indexing assigned by CRD

Subject index terms
Ethiopia; Family Planning Services; Female; Humans; Postpartum Period; Sex Education

Date of registration in PROSPERO
28 April 2020

Date of first submission
22 November 2019

Details of any existing review of the same topic by the same authors

Stage of review at time of this submission
The review has not started

Stage	Started	Completed
Preliminary searches	No	No
Piloting of the study selection process	No	No
Formal screening of search results against eligibility criteria	No	No
Data extraction	No	No
Risk of bias (quality) assessment	No	No
Data analysis	No	No

Revision note
I didn't make major change to the registered protocol. Just two minor issues: 1) I revised data of anticipated or actual review start date from 30 November 2019 to 16 April 2021 and Anticipated review completion date from 30 December 2019 to 16 October 2021. I revised theses dates because it has been a long time since I planned review but I did.t start yet. Now, I want to start review to include eligible studies conducted before 16 April 2021. 2) I changed my co-assessor to MKA as it is not convenient for KHB.

The record owner confirms that the information they have supplied for this submission is accurate and complete and they understand that deliberate provision of inaccurate information or omission of data may be construed as scientific misconduct.

The record owner confirms that they will update the status of the review when it is completed and will add publication details in due course.

Versions

28 April 2020
08 December 2021
