# Supplementary material for: Factors associated with modern contraceptives uptake during the first year after birth in Ethiopia: A systematic review and meta-analysis
Source: PLoS One. 2023 Feb 7;18(2):e0270055. doi: 10.1371/journal.pone.0270055 (PMC9904466; doi:10.1371/journal.pone.0270055)
Supplement: S1 Table — (DOCX) [file pone.0270055.s003.docx]

S1 Table. Search method used and Search Results from PubMed

**Research Question:** What factors are associated to postpartum contraceptive uptake in Ethiopia?

| Concept Number | Search concepts ( a combination of MeSH & key words) | Search Results |
| --- | --- | --- |
| #1 | "Contraception"[Mesh] OR Contraception[tw] OR “Contraceptive method*”[tw] OR “Family planning”[tw] OR “Family planning method*”[tw] | 79,831 results |
| #2 | "Postpartum Period"[Mesh] OR Postpartum*[tw] OR “Postpartum period”[tw] | 116,182 results |
| #3 | factor*[tw] OR “associated factor*”[tw] OR determinant*[tw] OR “factors influencing”[tw] | 6,006,010 results |
| #4 | "Ethiopia"[Mesh] OR Ethiopia*[tw] | 21,819 results |
| #5 | **#1 AND #2 AND #3 AND #4** | 61 results |

**Searches Limited by (Filtered by):**

- **#1 AND #2 AND #3 AND #4-** 61 results
- Full texts(reduced to56 from 61)
- Journal articles( Reduced to 55-from 56 above)
- Studies conducted on Human( Reduced to 32 from 55 above)
- Studies conducted on female( Reduced to 31 from 32 above)
- Ethiopia
- English
- Observational studies: cross-sections, comparative cross-sections, matched and unmatched case control, prospective and retrospective cohort studies
- 31 articles are selected at this level which is subjected for further selection after critical review.
