# Supplementary material for: Factors associated with modern contraceptives uptake during the first year after birth in Ethiopia: A systematic review and meta-analysis
Source: PLoS One. 2023 Feb 7;18(2):e0270055. doi: 10.1371/journal.pone.0270055 (PMC9904466; doi:10.1371/journal.pone.0270055)
Supplement: S3 Table — (DOCX) [file pone.0270055.s005.docx]

S3 Table. Data Extracted from the included studies by the variables

1. Women education

| Study | Formal education | | No formal education | |
| --- | --- | --- | --- | --- |
|  | Event | Total | Event | Total |
| Gejo NG et al.,2019 | 262 | 343 | 12 | 25 |
| Ashebir W. et al,2020 | 75 | 276 | 66 | 405 |
| Abraha TH. et al,2017 | 256 | 510 | 27 | 80 |
| Dagnew GW. et al,2020 | 332 | 974 | 215 | 1330 |
| Emiru AA. et al, 2020 | 353 | 457 | 409 | 824 |
| Wassihun, B. et al, 2021 | 171 | 437 | 9 | 30 |
| Nibret Mihretie G. et al, 2020 | 190 | 318 | 27 | 84 |
| Getachew Andualem Belete. et al, 2019 | 226 | 379 | 8 | 21 |

1. Sex resubmission

| Study | Sex resumed | | Sex not resumed | |
| --- | --- | --- | --- | --- |
|  | Event | Total | Event | Total |
| Gejo NG et al.,2019 | 258 | 302 | 14 | 66 |
| Abraha TH. et al,2017 | 264 | 403 | 19 | 187 |
| Berta M. et al, 2018 | 155 | 262 | 30 | 141 |
| Teka TT. et al, 2018 | 267 | 556 | 7 | 47 |
| Tafa L. et al, 2018 | 412 | 507 | 37 | 118 |
| Jima GH. et al, 2020 | 34 | 164 | 140 | 996 |
| Getachew Andualem Belete. et al, 2019 | 127 | 180 | 107 | 220 |

,

1. Menses Returned

| Study | Menses Returned | | Menses not returned | |
| --- | --- | --- | --- | --- |
|  | Event | Total | Event | Total |
| Gejo NG et al.,2019 | 243 | 287 | 29 | 81 |
| Ashebir W. et al,2020 | 86 | 272 | 55 | 409 |
| Abraha TH. et al,2017 | 156 | 205 | 127 | 385 |
| Taye EB et al, 2018 | 229 | 300 | 115 | 246 |
| Dona A. et al, 2018 | 186 | 484 | 31 | 200 |
| Berta M. et al, 2018 | 114 | 163 | 71 | 241 |
| Abera Y. et al, 2015 | 270 | 373 | 70 | 330 |
| Gebremedhin, A.Y. et al, 2018 | 266 | 361 | 379 | 442 |
| Tafa L. et al, 2018 | 334 | 421 | 115 | 204 |
| Nibret Mihretie G. et al, 2020 | 127 | 167 | 90 | 235 |
| Getachew Andualem Belete. et al, 2019 | 158 | 219 | 76 | 181 |

1. Discussing FP methods with partner

| Study | FP discussed | | FP not discussed | |
| --- | --- | --- | --- | --- |
|  | Event | Total | Event | Total |
| Ashebir W. et al,2020 | 80 | 267 | 61 | 414 |
| Dona A. et al, 2018 | 152 | 396 | 65 | 288 |
| Kebede Tefera. et al, 2020 | 133 | 295 | 14 | 86 |

1. ever heard about modern FP methods

| Study | Heard | | Didn’t Hear | |
| --- | --- | --- | --- | --- |
|  | Event | Total | Event | Total |
| Ashebir W. et al,2020 | 139 | 596 | 2 | 85 |
| Berta M. et al, 2018 | 173 | 341 | 12 | 63 |
| Tafa L. et al, 2018 | 447 | 617 | 2 | 8 |
| Nibret Mihretie G. et al, 2020 | 131 | 229 | 60 | 139 |
| Jima GH. et al, 2020 | 151 | 624 | 23 | 536 |

1. Counseling on PPFP during antenatal care

| Study | Counseled | | Not Counseled | |
| --- | --- | --- | --- | --- |
|  | Event | Total | Event | Total |
| Abraha TH. et al,2017 | 217 | 322 | 66 | 268 |
| Tafa L. et al, 2018 | 237 | 302 | 212 | 323 |
| Tafere, T.E. et al, 2018 | 72 | 187 | 85 | 636 |
| Jima GH. et al, 2020 | 161 | 592 | 13 | 569 |
| Getachew Andualem Belete. et al, 2019 | 97 | 127 | 137 | 273 |

1. Having ANC follow up

| Study | Had ANC follow up <4times | | having ANC follow up >4 | |
| --- | --- | --- | --- | --- |
|  | Event | Total | Event | Total |
| Abraha TH. et al,2017 | 18 | 55 | 262 | 524 |
| Dagnew GW. et al,2020 | 202 | 775 | 281 | 820 |
| Dona A. et al, 2018 | 177 | 456 | 40 | 228 |
| Teka TT. et al, 2018 | 151 | 320 | 111 | 172 |
| Abera Y. et al, 2015 | 330 | 629 | 10 | 74 |
| Emiru AA. et al, 2020 | 372 | 770 | 390 | 511 |
| Tafa L. et al, 2018 | 443 | 603 | 6 | 22 |
| Wassihun, B. et al, 2021 | 166 | 365 | 15 | 43 |
| Getachew Andualem Belete. et al, 2019 | 133 | 241 | 101 | 159 |

1. Having PNC follow up

| Study | Had PNC follow up | | Not having PNC follow up | |
| --- | --- | --- | --- | --- |
|  | Event | Total | Event | Total |
| Abraha TH. et al,2017 | 173 | 258 | 110 | 332 |
| Dona A. et al, 2018 | 175 | 500 | 42 | 184 |
| Teka TT. et al, 2018 | 220 | 340 | 54 | 263 |
| Mengesha ZB. et al, 2015 | 19 | 51 | 74 | 848 |
| Abera Y. et al, 2015 | 107 | 185 | 233 | 518 |
| Emiru AA. et al, 2020 | 172 | 192 | 590 | 1089 |
| Tafere, T.E. et al, 2018 | 96 | 159 | 61 | 664 |
| Seifu B. et al, 2020 | 98 | 186 | 46 | 168 |
| Nibret Mihretie G. et al, 2020 | 52 | 67 | 165 | 335 |
| Getachew Andualem Belete. et al, 2019 | 116 | 162 | 118 | 238 |
| Jima GH. et al, 2020 | 132 | 520 | 42 | 640 |

1. Having experiencing of contraceptive use before last pregnancy

| Study | Had FP experience | | No FP experience | |
| --- | --- | --- | --- | --- |
|  | Event | Total | Event | Total |
| Ashebir W. et al,2020 | 113 | 485 | 28 | 196 |
| Taye EB et al, 2018 | 302 | 451 | 42 | 95 |
| Gebremedhin, A.Y. et al, 2018 | 557 | 629 | 88 | 174 |
| Seifu B. et al, 2020 | 121 | 249 | 23 | 105 |
| Getachew Andualem Belete. et al, 2019 | 154 | 242 | 80 | 158 |

1. Gave birth in the facility

| Study | Gave birth in the HF | | No Facility birth | |
| --- | --- | --- | --- | --- |
|  | Event | Total | Event | Total |
| Dagnew GW. et al,2020 | 350 | 938 | 197 | 1366 |
| Mengesha ZB. et al, 2015 | 57 | 170 | 36 | 729 |
| Like Berhanu Kenea. et al, 2021 | 353 | 429 | 56 | 168 |

1. Women Marital status

| Study | Married | | Not married | |
| --- | --- | --- | --- | --- |
|  | Event | Total | Event | Total |
| Taye EB et al, 2018 | 337 | 519 | 7 | 27 |
| Gebremedhin, A.Y. et al, 2018 | 627 | 748 | 18 | 55 |
| Wassihun, B. et al, 2021 | 161 | 345 | 20 | 63 |

1. length of time after last delivery

| Study | <6 months | | >6 months | |
| --- | --- | --- | --- | --- |
|  | Event | Total | Event | Total |
| Berta M. et al, 2018 | 94 | 264 | 91 | 140 |
| Abera Y. et al, 2015 | 93 | 291 | 247 | 412 |
| Gebremedhin, A.Y. et al, 2018 | 192 | 271 | 453 | 532 |
| Nibret Mihretie G. et al, 2020 | 87 | 198 | 130 | 204 |
| Kebede Tefera. et al, 2020 | 75 | 232 | 72 | 149 |
| Gejo NG et al.,2019 | 55 | 101 | 220 | 270 |
